# The Collision Between the Classroom Voice(s) and the Voice of the Mainstream Culture on End-of-Life to Cultivate Students' Attitudes Toward Death in China

**DOI:** 10.3389/fpsyg.2022.879787

**Published:** 2022-06-22

**Authors:** Ling Meng, Li Yi, Tian Li

**Affiliations:** ^1^School of Foreign Studies, South China Normal University, Guangzhou, China; ^2^School of Foreign Languages, Sun Yat-sen University, Guangzhou, China; ^3^School of Basic Medicine, Fourth Military Medical University, Xi'an, China

**Keywords:** voice, polyphony, attitudes toward death, end-of-life, ethnography, classroom discourse

## Abstract

Using Bakhtin's notion of polyphony, this study explored the discussion of the end-of-life issues in the *Course on Life and Death Education* in one Chinese university. Ethnographic methods were adopted to investigate the collision between the classroom voices and the voices of the mainstream culture on end-of-life in the process of developing students' attitudes toward death. The findings revealed that “to understand death” involved challenging the voice of “strangeness and fear of death”; “honestly facing up to and accepting the feelings of the fear, pain, and helplessness” was the response to “be brave”; and the goal “to die peacefully” resisted the notion of “extending life at any cost.” Through the collision between these voices, students developed their attitudes toward death in facing, understanding, accepting, and choosing how to die. The analysis further revealed that providing only one “answer” to death by the teacher is not sufficient or effective to foster students' attitudes toward death because the students are a diverse group holding different views on the end-of-life issues, which demonstrated the importance of creating dialogues in the life and death education.

## Introduction

### Background

In the past, death was commonly seen in normal life. People did not seek to deny or avoid death but learned to accept it by continuously facing dying and dead family members, as well as their funerals, as a natural part of their own life (Lakotta and Schels, 2004; Xi and Wang, 2011). Death was thus not mysterious. However, nowadays, with the development of medical technology, death has changed in both space and time: the space of dying has moved from the home to the hospital and the time of dying has lengthened from a short moment to a long period of time (Lu, 2016). Death has been redefined as the time when a machine is switched off rather than as the end of the body's self-destruction process, and the experience of how to die has also been reconstructed (Xi and Wang, 2011). Lately, it is relatively easy for people to avoid facing pain and death, and death is becoming increasingly mysterious. People's understanding of death is becoming growingly vague, and people's fear of death is becoming progressively intense (Lakotta and Schels, 2004) Furthermore, as one of the important issues surrounding “death,” the process of end-of-life is the one that we are increasingly unwilling to touch because it makes us feel depressed. However, avoiding this issue, on the one hand, leads to regrets on the part of the dying when they find themselves unable to leave the world peacefully, and it, on the other hand, makes their family members experience confusions and losses (Yuan, 2017). This study, therefore, aims to explore the end-of-life issues in the *Course on Life and Death Education* in one University in China, a course which is only a recent offering.

### The Practice and Research of Death Education

The concept of death education refers to the education of knowing about death and how to deal with death, dying, and mourning (Corr et al., 2019). It originated in the United States with the rise of “Death Awareness Movement” in the 1960s. Death education had, thereafter, become popular in Britain, Ireland, and Japan, and it has gone through a transition from the discussion of the relationship between life and death to the curriculum development in death education, as well as the humanization of the dying process (Doka, 2015). In the United States, thanatology, a special subject area that studies the behaviors, thoughts, emotions, and phenomena related to death and dying had gradually emerged as an independent specialization with a greater understanding of death and the implementation of death education (Wass, 2004). Thanatology is an interdisciplinary field ranging across medicine, biology, humanities, and social sciences, and it seeks an active dialogue between science, technology, and humanities (Wang et al., 2007). The subject first developed in the fields of medicine, nursing, and psychological counseling, and it encouraged people to set up death education courses from kindergartens to universities (Bailis and Kennedy, 1977; Bowie, 2000; Lee et al., 2009; Doka, 2015; Pitimson, 2021). The instruction methods of these courses are mainly didactic with a focus on the cognition and perception, or are experiential with a focus on the feeling and reflection (Durlak and Reisenberg, 1991).

With the development of the practice of death education, the research on death education had developed from the exploration of its definition, significance, content, goals, and curriculum design to the study of its course effects (e.g., Corr, 2016), such as using questionnaires or experiments, to evaluate the effect of death education curricula on reducing anxiety around death (e.g., Leviton and Fretz, 1979; Knight and Elfenbein, 1993; Maglio and Robinson, 1994). The focus of the research on attitudes to death had moved from the negative attitudes, such as fear, anxiety, escape, and denial (e.g., Templer, 1970), to the positive attitudes (e.g., Klug and Boss, 1976), and then to the understanding of the complexity of attitudes to death (e.g., Holcomb et al., 1993; Wong et al., 1994). The exploration has focused on the factors affecting attitudes to death on multiple levels, such as gender, age, educational level, religious belief, and identity at the individual level, on the experience of death and talking about death at home at the environmental level, and on the impact of death education on attitudes toward death at the educational level. Research on the influential factors at the educational level is consistent with the focus on curriculum effects in the field of life and death education. The life and death education had been proved to exert a positive impact on people's attitudes to death, including changing students' view of death (e.g., Smith-Cumberland and Feldman, 2006; Kim and Lee, 2009; Wong, 2017), helping students to treat death as a natural process (e.g., Leviton and Fretz, 1979), and reducing students' anxiety about death (e.g., Knight and Elfenbein, 1993; Maglio and Robinson, 1994; McClatchey and King, 2015).

The topic of “death” is still a taboo in China. Traditional Chinese Confucianism believes that “we cannot understand death without knowing life” and emphasizes the importance of living more than the death and dying. Hence, death education is mostly offered in the form of life and death education or life education in China (Zhang and Wang, 2015). Death education, life and death education, and life education are the three different expressions for the same term (Tang, 2004). This study uses “life and death education” for death education, referring to the education of “living facing death.” The term “life and death education” conforms to the Chinese tradition to highlight living and to emphasize the integration of life and death (Zhang, 2001; Fu and Xu, 2006). Life and death education in China only has a short history of about 30 years. Although it has developed rapidly into a successful practice in Taiwan and Hong Kong of China, it remains premature in Chinese Mainland (Xie and Xu, 2007). Only a few universities or medical colleges offer the life and death education courses, and the scientific and systematic life and death education system is under construction.

The life and death education courses in China mainly use a combination of lectures of theory and case studies, and the teacher-centered way of teaching is prevailing (Lu, 2016). A few courses integrate students' sharing and experiences to allow students' independent choices, but students' choices of topics only produce a sporadic coverage of the topic of death (Li, 2013). Research on life and death education has mainly dealt with its necessity, goals, content, and different approaches, such as palliative care, prevention of adolescent suicide, and such like (e.g., Zhuang, 2002; Ping and A, 2005; Wang and Li, 2020), but empirical studies are rarely seen. A few empirical studies have explored the necessity for life and death education by using questionnaires on attitudes toward death and the demand for life and death education (Lin et al., 2012), which, however, remain largely in the realm of theoretical exploration. Research on end-of-life in life and death education has paid more attention to attitudes toward death, using questionnaires or experiments to test the impact of life and death education on college students' attitudes toward death (e.g., Li et al., 2017). Few qualitative studies on life and death education had been conducted both in China and abroad, and ethnographic research on courses in life and death education is quite scarce. Further exploration is thus necessary, which can provide a practical basis for the development of life and death education, build an empirical basis for research in life and death education, and promote the development of both practice and theory of life and death education.

### Classroom Discourse in Life and Death Education

Death discourse refers to the topics related to death and dying and the discourse used to discuss these topics, including the oral narration and dialogue of the experience about death and the representation of death in text and film (Tsai, 2010). The study of death discourse has increasingly attracted the attention of the researchers from the fields of literature, linguistics, education, psychology, sociology, and medicine (Wang, 2016). However, the study on the death discourse in life and death education is so far quite limited.

Death discourse in life and death education course is not only a discourse to discuss death but also a part of educational discourse with educational significance. Previous studies on classroom discourse worldwide have mostly focused on the language classroom or the mainstream classroom for students from different languages and cultures with most of their attention paid to the interaction mode, teacher discourse, student discourse, and the power in discourse (Hei, 2013). However, the classroom discourse in life and death education has not yet been addressed adequately.

### Theoretical Framework and Research Questions

Bakhtin (1984) notion of “polyphony” was adopted as the fundamental guiding concept for this study. The metaphor of polyphony is originally from music, where it refers to more than one melody integrated into a combined texture, and it is subsequently used to refer to various voices and opinions in literary works. This study employed this concept in life and death education to refer to the two or more voices simultaneously present in the discussion of death issues. Bakhtin defines the voice as “a synthesis of one's thoughts, opinions, and attitudes expressed through language” (Bakhtin, 1998 p. 3). Voices can be identified in the dialogue between different subjects and in the reflection within one subject (refer to Table 1). For example, in exchange III, while the viewpoints of students 8 and 12 are different, there are two different voices from two subjects; while student 10 expresses two different viewpoints, there are two different voices within the same subject. Different voices are thus distinguished from different viewpoints. The dialogism between voices is understood as “a special form of interaction between different consciousnesses with the same value” (Dong, 1994 p. 7), which is not just the presence of different voices but how they respond to each other. Similar to musical polyphony, it is the “counterpoint” of different voices with each other that needs to be identified to understand the voices constructed in the classroom. These voices demonstrate the challenges, responses, and resistance among the voices and the development of attitudes toward death.

**Table 1:** The voices constructed on end-of-life in the classroom.

**Transaction**	**Exchange**	**Foci**	**Subjects of voices**	**Viewpoints**
Discussion	I	Who has been in the hospital? How do you feel about the hospital?	Student 1	Doctors, nurses, injections and medicines
			Student 2	Panic and nervous
			Student 3	Reflect the themes of human world
			Teacher	The movie chip will bring us to experience hospital from the perspective of a patient
		What is your feeling after watching the clip of *Wit*? (Vivian's medical check and treatment for cancer)	Student 4	Vivian followed doctors like a submissive child
			Student 5	They did not treat her as a human being
			Student 8	Vivian became an illness case
			Student 6	Vivian is calm and not afraid of death, but cooperatively to accept the treatment
			Teacher	As student 8 mentioned a key issue, which is extremely sharp in China. When doctors facing such a large workload, whether they still have the ability to treat patients as human beings or they can only treat as patients; from traditional medicine to modern medicine great changes have taken place, such as from one to one to one to more, from experience to technique, from man to machine, with the separation of information, technology and knowledge, human, emotion and humanistic care are separated
	II	When facing end-of-life, what can we learn from these two scenes? (Accompany the dying)	Student 8	Treat patient gently
			Student 7	Use body language to comfort patients
			Student 8	Slow, soft and rhythmic touch
			Student 6	Respect for life is not to extend life
			Student 10	Accept Vivian's feelings
			Student 11	Not to say be brave, not to say you will be fine to end-of-life patients
			Teacher	Use body language to express care and comfort; co-frequency breathing; accept Vivian's feeling; talk about deathbed preferences honestly and respect those choices; as a companion, you need to settle your heart first; five stages of end-of-life
	III	A series of if questions after watching the clip of *Wit* (Vivian's end-of-life):	
		If you were Vivian, what kind of choice would you make? Why?	Student 8	I would choose not to be rescued, I would choose to go home to die there peacefully
			Student 12	I would choose to rescue, because there are things I cannot give up
			Student 13	I would choose to rescue, because I do not want to hurt my family
			Student 14	I have no idea at all, it is a hard decision, I still need more time to think
		If that were your parents, what kind of choice would you make? Why?	Student 10	A difficult decision to be made. I could not bear to give up. The scene of rescue in the film looks like painful, which makes me could not bear to make them suffer
			Student 15	If there was no cure, I would choose not to rescue
			Student 6	First thought is to rescue, but I think I would follow my parents' willingness. If I do not know their choices, I will choose to rescue
			Student 9	I will ask my parents several times and follow their choices
			Student 16	If I chose not rescue them, I would under the great public pressure. It will be considered as an unfilial behavior
		If your parents made the choice, what do you think they would choose? Why?	Student 17	I think my parents will choose not to be rescued, because they want to die with dignity
			Student 18	I think my parents will choose not to be rescued, because they do not want to become my burden
			Student 10	I think my parents will choose not to be rescued, just let nature take its course
			Student 19	I think my parents will choose to be rescued, because they want to accompany me as long as they can
			Student 20	My parents have already told me they would choose not be rescued, because they chose to donate their organs
			Teacher	I will find a time to play a Taiwanese movie about body donation, which I feel greatly touched
Lecturing		Reading: medicalization of death	Teacher	Death is becoming the time of switching of the machine; good death; die with dignity; living will

This study aims to explore the end-of-life issues in the *Course on Life and Death Education*. The voice of traditional mainstream Chinese culture is that people should try their best to extend the life of the elderly to show their filial obedience. This study focuses on the collision between the classroom voices and the voices of the mainstream culture in the process of students' development of their attitudes toward death. The research questions guiding the study are as follows:

1. What are the voices constructed in the classroom?
2. How do the classroom voices and the voices of the mainstream culture contrast with each other?
3. In the collision between the voices, what kind of attitudes toward death are developed?

## Methodology

### Design

An ethnographic methodology (Hammersley and Atkinson, 2007) was adopted to investigate the voices constructed in the classroom on end-of-life issues and the collision between the classroom voices and the voices of the mainstream culture in developing the students' attitude toward death.

### Research Field and Research Participants

To build up a sample that is satisfactory for the specific needs to investigate the University course of *Life and Death Education* in China, purposive sampling (Cohen et al., 2007) was employed in the selection of *Course on Life and Death Education*. It is a one-semester two-credit elective course of liberal education offered at a Chinese prestigious comprehensive University in Beijing, which provides education on basic disciplines in the sciences and humanities, as well as teacher education and educational science. The objective of this course is to help college students to understand death and guide them to explore how to live out the meaning of their lives through the in-class discussion of death-related issues. The teachers who taught this course, together with the 35 undergraduate students of different years and different majors^1^ participating in this course, were invited and voluntarily participated in this study. The teacher is a retired part-time teacher who has rich working experience in mass media, psychological consultation, and mental health education. All the students who chose this course are born after the year 1995 and the ratio of male-to-female students is about 1:5.

### Data Collection and Data Analysis

The dataset includes the follows:

1. classroom participation observation (3-h observation per week for 12 weeks, audio-recorded classroom teaching and discussion with the permission of the participants, and field notes);
2. in-depth interview with teacher and students (approximately 1-h individual face-to-face interview per person about the reasons why they chose the course, their experiences and feelings in the course, and their gaining and suggestions for this course); and
3. supplementary documents (e.g., course materials, readings, and assignments).

This article is a part of the research project of *Polyphony of Death: An Ethnographic Research on a Life-and-Death Education Course for University Undergraduates*. To fulfill the needs of investigating end-of-life issues, the data mainly draw on the classroom observation, the audio recordings of the class, and the course materials from week 8 of the course. The course materials from week 8 include a 21-min film clip from *Wit*^2^ and an article about the discussion of end-of-life choices and dignity entitled *Dialogue with ICU: The Boundlessness of Life and Death - The End of Life in the Age of Technology and the Meaning of Death* (Xi and Wang, 2011).

After transcribing the classroom discourse on end-of-life issues by the model of the Birmingham School (Sinclair and Coulthard, 1975), the analysis of the classroom discourse examined the exchange patterns in the verbal interactions among the teacher and students, the transactions within a lesson, and the foci of the transactions. The analysis focused on the different voices, the dialogue among voices, and the attitudes toward death that had developed in the discussion process.

The original data were in Chinese. We did all our analyses based on the Chinese transcripts. Only those experiences related to the interests of the study were selected, translated, and presented in English in the Section “Results” by us to prevent any valuable meanings from being lost in the process of translation. Since we use extensive quotes from the participants' own voices to present our data, one of the major tasks has been in relation to the translation of direct quotes. We used a combination of literal and free translation, although the decisions for either the literal or free one were not easy ones, because there was a tension between readability and authenticity (Birbili, 2000). Moreover, two of us discussed the translation-related issues raised and checked all the translations to reach the final agreement.

### Ethics

Informed consent was obtained from each participant for both the recordings of classroom discussion and the use of the excerpts from the course materials. When analyzing and presenting the classroom discourse, the real names of the teacher and students were replaced by the terms of “teacher” and “student” and the students were referred to by different numbers, such as student 1 and student 2.

## Results

### Course Background

The film clip of *The Wit* was used throughout the teaching in *The Course on Life and Death Education* to facilitate the joint exploration of the issues around death among the teacher and students. Such issues were rarely articulated or discussed in daily life similar to the other similar issues of suicide, bioethics, end-of-life, funeral etiquette, and so on. From the perspective of existentialist psychology, the course took the thanatology as the main focus and the life growth of college students as the secondary focus in interpreting the issues of life and death. The aim was to help the students, through this course, think about how to live a meaningful life (teacher's interview).

The classroom used for this course was a traditional Chinese classroom and the classroom was divided by a raised platform into the teacher's zone and the students' zone. In the students' zone, the desk and chair sets were placed closely together with eight sets in each row and two on the left and right sides and four in the middle (field notes). This kind of classroom is suitable for a large class size and teacher-centered way of teaching but is not ideal for discussion-based interactive classes.

Following the Birmingham School framework, four types of transactions were identified in the classroom discourse, namely, lead-in (3%), film-clip watching (12%), discussion (60%), and teacher's lecturing on main knowledge points (25%)^3^. The class was characteristic of a high proportion of the class discussion time. The overall structure of the discussion exchange was similar to the Birmingham school's typical three-move exchange, namely, teacher's *initiation*, students' *response*, and teacher's *feedback* (IRF) (Sinclair and Coulthard, 1975). But commonly there were more than three moves in an exchange. The biggest difference lay in the teacher's feedback. Under the rule of “no single right-or-wrong standard,” the teacher employed non-evaluative means to encourage multiple voices from the students. While she did not avoid explicitly stating her own views, she did not single out her own views for students as the only acceptable viewpoints (Meng, 2017) (refer to section (1) and (2)).

### The Voices Constructed in the Classroom

The analysis of the discussion on the end-of-life issues in the *Course on Life and Death Education* found that multiple voices had come out in the classroom (refer to Table 1). Through the dialogue between these multiple voices, the main themes of end-of-life construed by the voices in the classroom were “to understand death,” “to honestly face and to accept the feelings of fear, pain, and helplessness,” and “to die peacefully.”

As shown in Table 1, the exchange I functioned as a lead-in to the discussion on end-of-life. The discussion of the end-of-life issues then centered on learning how to accompany the dying (exchange II), how we are going to die (exchange III), and how to die peacefully (lecturing transaction).

The voice of “to understand death” was constructed through the film clip and through the classroom dialogue reflecting on one's behavior and choices. At present, most people die in hospitals. Due to various restrictions, we seldom have the chance to see a person in the process of dying or their actual death. In the exchanges I to III, according to the movie clips, a 48-year-old English literature professor, named Vivian, was suffering from Stage IV ovarian cancer undergoing a medical check and being treated for cancer (3 min). She engaged in a midnight conversation with nurse Susie (8'30”) and she was visited by her PhD supervisor Ashford, who read *The Runaway Bunny* for her, and Vivian was falling asleep in her arms (7'). Her end-of-life (2'30”) was used in order to give students the chance to confront death and dying. Watching the film clip was an opportunity for the students to demystify death gradually and to understand the dying process. Then, from the very specific dying scene shown in the film clip, the teacher raised questions to discuss with students to help them to face and to understand death.

Under the umbrella of “to understand death,” the voice of “to honestly face and to accept the feelings of fear, pain, and helplessness” was constructed through the discussion in exchange II. After watching two scenes of people warmly accompanying the dying person in the movie clip, the teacher and students discussed what they could learn from facing the situation of the end-of-life issues. Refer to the discussion in section (1) below:

### (1)

Teacher: *When facing end-of-life, what can we learn from these two scenes?*

…

Student 7: *I have learned that we can* ***use body language to comfort patients**, such as nurse Susie's touch and supervisor Ashford's embrace in the film*.

Teacher: *Great! Use body language to express our care and consolation*.

…

Student 10: *To* ***accept Vivian's feelings*** *is an important thing for me to learn. For example, Vivian told Susie she was awake and she just kept thinking and she could not figure things out. Susie told her what she was doing was really hard and it seemed like it was out of her control. Vivian said she was scared. Susie replied oh dear, sure. Moreover, when she told Ashford she felt so bad, Ashford said yes, I knew you did. I could see it*.

Teacher: *Yes, to accept Vivian's feelings*.

…

Teacher: *OK, I will talk about what I have learnt from it. Firstly, as what you have mentioned just now*, ***use body language to express care and comfort**. Moreover, I learned in the palliative volunteer training that apart from body language, we can also use* ***co-frequency breathing**. With a patient who maybe will not be able to talk and so the two of us will not have much verbal communication, I can try to keep the same frequency of breathing with this patient to make them feel comfortable. Secondly and importantly, I learned to* ***accept Vivian's feelings*** *of fear and loneliness, as some of you said. When we say “be brave and you'll be fine,” it shows that we are in a state of refusal. Thirdly, I have learned to* ***talk about end-of-life preferences honestly and respect those choices**. In the film, Susie asked Vivian's preferences about whether she wanted to be revived. Fourthly, it is very difficult to be a companion, you need to* ***be at peace in yourself*** *first…*

The above example shows that in the discussion “to use body language to comfort patients” and “to accept Vivian's feelings” construct the voice of “to honestly face and to accept the feelings of fear, pain, and helplessness.” In addition, the convergence of the voices of teacher and students in dialogic exchange further emphasized this voice.

Similarly, under the umbrella of “to understand death,” the voice of “to die peacefully” was constructed through the discussion in exchange III and the lecturing transaction. After watching Vivian's end-of-life scene in the movie clip, teacher and students discussed three “if” questions, namely, first, if you were Vivian, what kind of choice would you make and why? Second, if Vivian were your parent, what kind of choice would you make and why? Third, if a parent of yours had to make this choice, what do you think they would choose and why? As shown in Table 1, the students tended to make a range of choices. For the first “if” question, while the majority of students chose to refuse revival, a minority of them chose to revive and a few had not made up their minds yet. For the second “if” question, under the scenario that they did not know their parent's wishes, the students struggled to make a decision. On the one hand, they could not bear to give up the chance (that their parents might be revived). On the other hand, they could not bear for their parent to suffer either. However, if they chose to refuse the revival, they would be under great pressure from the public opinion which would consider them unfilial children. For the third “if” question, the majority of students thought their parents would choose not to be revived, because they would not wish to become a burden on their children. A minority of them thought that their parents would choose to be revived, because they wanted to stay with them. A few of them already knew that their parents had chosen not to be revived because they had indicated they would donate their organs. These different choices are shown in Section (2) below:

### (2)

Teacher: *If you were Vivian, what kind of choice would you make? Why?*

Student 8: *I would choose* ***not to be revived**; I would choose to go home* ***to die there*** ***peacefully***.

Teacher: *I see*.

…

Teacher: *If that were your parent, what kind of choice would you make? Why?*

Student 10: *It's a difficult decision to make. I could not bear to give up on them*.

*But* ***the scene of revival in the film looks painful**, which* ***makes me feel I could not bear*** ***to have them suffer***.

Teacher: *It's all tangled up*.

…

Teacher: *If your parents made the choice, what do you think they would choose? Why?*

Student 17: *I think my parents will choose* ***not to be revived**, because they would want* ***to*** ***die with dignity***.

Teacher: *All right, thank you*.

…

In (2), the student voices “not to be revived,” “to die there peacefully,” “the scene of rescue in the film looks painful, which makes me feel I could not bear to have them suffer,” and “to die with dignity” echoed with the voice of “to die peacefully.”

Furthermore, the divergence between the voices of the teacher and students in dialogic exchange highlighted the voice “to die peacefully.” In order to help the students to discuss their end-of-life choices, the teacher guided them to read an article entitled *Dialogue with ICU: The Boundlessness of Life and Death - The End of Life in the Age of Technology and the Meaning of Death* after watching the emotional revival scene in the film. In this discussion, the teacher pointed out another possibility for death, i.e., death with dignity, which was different from dying alone or painfully in the hospital. The notion of death with dignity refers to the choices that are available for the people near the end of incurable illness to refuse revival and the help of life support systems and to let death come naturally, neither ahead of time nor behind time. In this process, family should try their best to respect and realize the dying person's wishes to the maximum extent and to allow them to bid farewell to the life with dignity (Living Will Promotion Association, 2015). From the viewpoint of death with dignity, death is regarded as a normal part of the process of individual growth, which rewrites the definition of death in the age of technology as “shutdown time.” The teacher then shared her feelings about her visit to the hospice wards in Taiwan compared to the experience of those who had experienced palliative care in China.

### (3)

Teacher: …*The ward looks like this: it is very bright, like home, with reception area, etc. In such a place where death wandered, I was reluctant to leave. I did not feel the slightest fear. Instead, I saw a lot of smiling faces and heard a lot of laughter. The director of the hospital told me that many people would go home after they have brought their pain under control here, so as to give them the last time with their families. In China, some of my friends were navigating palliative care, which they felt pretty good about. The mother of one of my friends ended up dying under the care of her family, they were able to say a lot to each other and eventually she died peacefully…*

There is a sharp contrast between the scene described by the teacher and the scene of death coming after the shocking revival by modern medicine in the film. When Doctor Jason found that Vivian was unconscious, he quickly informed the cardiopulmonary resuscitation (CPR) team. Before their arrival, he jumped onto Vivian's bed and pulled open her coat to apply CPR and mouth-to-mouth resuscitation. Later, the CPR team conducted cardiac defibrillation. In the process described by the teacher, the ward was surrounded by calm and warmth, which dissolved the pain, the loneliness, and the horror of death. The voice of “to die peacefully” was constructed in the collision between these voices.

### The Collision Between the Classroom Voices and the Voices of the Mainstream Sulture

Compared with the strong voices of the mainstream Chinese culture, the voices constructed in the classroom represented a kind of supplement and confrontation, which reflected the polyphony around the end-of-life issues. The voices constructed in the classroom challenged, responded, and resisted the voices of mainstream culture: “to understand death” challenged the voice of “strangeness and fear of death”; “to honestly face and to accept the feelings of fear, pain, and helplessness” responded to the “be brave” voice; and “to die peacefully” resisted the voice “extend life at any cost” (refer to Table 2).

**Table 2:** The collision between the classroom voices and the voices of the mainstream culture.

**The voices of the mainstream culture**	**The voices constructed in the classroom**
Strangeness and fear of death	To understand death
Be brave	To face up to and accept the feelings of the fear, pain, and helplessness
Extending life at any cost	To die peacefully

The voice of “to understand death” constructed in the classroom challenged the voice of “strangeness and fear of death” in mainstream culture. In the lead-in transaction, the students and the teacher analyzed the reasons why people are afraid of death, which mainly include the fear of unknown (e.g., the time of death), the fear of the known (e.g., the death is a bad thing), the fear of the miserable dying process, the issues after death, the worry of the unfinished business, and the concern for those who they love and care. Through the discussion about the reasons why people today are more afraid of death than in the past, they further explored the changes in the internal and external factors affecting the fear of death, e.g., the lack of faith and religion, the change from superstition to scientism, and the mysteriousness of death due to the temporal and spatial changes of death mentioned above. The discussion of these questions reflected the voice of “strangeness and fear toward death.” This voice is challenged by the voice of “to understand death” constructed in the classroom.

The voice of “to honestly face and to accept the feelings of fear, pain, and helplessness” that was constructed in the classroom had responded to the voice of “be brave” in mainstream culture. As seen in the discussion in exchange II, student 11 said she has learned not to say “be brave” to the dying (refer to Table 1). This voice was amplified in the teacher's feedback. The teacher said that when we tell a sick or dying person to be brave, it showed that we were in a state of refusal [refer to Section (1)]. Thus, the voice of “be brave” was contrasted with the voice of “to honestly face and to accept the feelings of fear, pain, and helplessness.”

The voice of “to die peacefully” constructed in the classroom resisted the voice of “extending life at any cost” in the mainstream culture. In the discussion in the exchange II, student 6 said that she has learned that respect for the life does not necessarily mean to extend life (refer to Table 1). In the Chinese cultural context, the voice of unconditional extension of life is very strong, and the voices constructed in the classroom had resisted the strong voice of showing filial obedience by the unconditional extension of life.

### The Development of Attitudes Toward Death

The students' attitudes toward death had been developed in the collision between voices in terms of facing, understanding, accepting, and choosing how to die.

In traditional Chinese culture, death is considered a taboo. However, the *Course on Life and Death* provided a platform for discussing the end-of-life issues around the topic of death. The film clip and the discussion in the class developed students' attitudes toward death in terms of facing up to death. The voice of “to understand death” constructed in the classroom developed students' attitudes toward death as understanding death (refer to the voices constructed in the classroom section). The voice of “to honestly face and to accept the feelings of fear, pain, and helplessness” constructed in the classroom developed students' attitudes toward death as accepting death. The voice of “to die peacefully” constructed in the classroom developed students' attitudes toward death as choosing how to die. In Section (3), after the teacher shared her feelings about her visit to the hospice wards in Taiwan, she continued to discuss her living will from the *My Five Wishes* website to explain what she can do as an individual.

### (4)

Teacher: My Five Wishes is a website of choice and dignity. I can choose to fill in what medical services I want or do not want, whether I want to use or not use life support systems, how I want others to treat me, what I want my family and friends to know, and who I want to help me. For example, in completing my living will I refused cardiopulmonary resuscitation, the use of a ventilator, the use of gastroesophageal intubation, blood transfusion, hemodialysis and so on. Although this document does not have legal effect at present, it can at least relieve the pressure on my family members. Do you remember the struggle when you were asked to choose whether or not to have your parents revived? This is something we are trying to push forward.

The use of a living will as mentioned by the teacher meant that we could even arrange our own death according to our preferences, thus developing students' attitudes toward death as choosing how to die.

## Discussion and Conclusion

In the exploration of the end-of-life issues in the *Course on Life and Death Education* with the help of movies and texts, it was found that the teacher opened up a diversity of voices. These voices broke through the teacher's single voice in the classroom with the teacher's teaching, which helped to build a platform for the flow of “human dialogue” between the teacher's single voice and to inner and more convincing voices of all the participants (Nesari, 2015). Through the dialogue between these multiple voices, the voices constructed around end-of-life in the classroom were “to understand death,” “to honestly face and to accept the feelings of fear, pain, and helplessness,” and “to die peacefully.” Students' attitudes toward death as facing, understanding, accepting, and choosing how to die were thus developed in the collision between voices. This course built a platform for students to explore the end-of-life issues around the topic of death and also provided a chance for students to reflect on their life and living (Corr, 2016). For a diverse group of college students who hold different views on end-of-life, it may not be efficient or effective to instill all kinds of attitudes toward death by providing one single “answer” by the teacher. The course which was observed in this study had made a good attempt to create a dialogue around the life and death education (Pitimson, 2021).

However, due to the large class size and time constraint, the dialogue in the class was mainly under the guidance of the teacher, and the dialogue between or among the students was not quite sufficiently realized. Moreover, in the exchange III, the three questions were not closely linked to the scene shown in the film clip. It would have been better to add a question about “what touched or moved you in the clip” before raising the three “if” questions. It would also seem like to worth trying to use movies throughout the teaching to lead students to face death. If conditions permit, more diverse ways would be introduced in the future, such as personal narratives or other ways, to reduce the distance between the students and death. The content relating to facing up to death oneself can be added as appropriate. How to explore and gain more of a three-dimensional picture of end-of-life issues in the classroom is worth further investigating. This research only audio-recorded the classroom teaching and discussion, and further study would video-record the observation process and use multimodal analysis methods to interpret data.

## Data Availability Statement

The original contributions presented in the study are included in the article/supplementary material, further inquiries can be directed to the corresponding author/s.

## Ethics Statement

The studies involving human participants were reviewed and approved by Ethics Committee of South China Normal University. The patients/participants provided their written informed consent to participate in this study.

## Author Contributions

LM, LY, and TL: conceptualizing and writing, reviewing, and editing the manuscript. LM: methodology, collecting data, and writing the original draft. LM and LY: analyzing data and translating the raw data presented in the results. TL: data analysis and member-checking. All authors contributed to the article and approved the submitted version.

## Funding

This study was supported by the National Social Science Foundation of China (19AYY001), the Humanities and Social Sciences Foundation of the Ministry of Education of China (21YJC740066), and the Guangdong Philosophy and Social Science Foundation (GD21CWY06 and GD19YYY08) and the Guangzhou Philosophy and Social Science Foundation (2022GZQN38). The funders had no role in the study design, data collection and analysis, and decision to publish or preparation of the manuscript.

## Conflict of Interest

The authors declare that the research was conducted in the absence of any commercial or financial relationships that could be construed as a potential conflict of interest.

## Publisher's Note

All claims expressed in this article are solely those of the authors and do not necessarily represent those of their affiliated organizations, or those of the publisher, the editors and the reviewers. Any product that may be evaluated in this article, or claim that may be made by its manufacturer, is not guaranteed or endorsed by the publisher.

## Footnotes

^1^Altogether there were more than 50 participants in the class, the auditors, the teaching assistant, and the present researchers were not included among the research participants.

^2^The website of the film *Wit*: http://www.tudou.com/programs/view/jKHU099T0u4/ (accessed 15 February 2022).

^3^The calculation of percentage: the time ratio of each transaction. The whole class is 180 min with a 10-min break.
